# Improving recognition and management of inpatient delirium in Parkinson’s disease: evidence review and implications for clinical care

**DOI:** 10.3389/fnagi.2025.1693827

**Published:** 2025-10-29

**Authors:** Colin Coleman, Adrianne Smiley, Sheera Rosenfeld, Anne Brooks

**Affiliations:** Parkinson’s Foundation, New York, NY, United States

**Keywords:** delirium, Parkinson’s disease, PD, diagnosis, prevalence, risk factors, prevention, treatment

## Abstract

Delirium is an acute disturbance of attention, arousal, and cognition that fluctuates in severity and is a common yet under recognized complication in hospitalized patients with Parkinson’s disease. In Parkinson’s disease, symptoms such as rigidity, bradykinesia, hallucinations, and slowed thinking overlap with the clinical features of delirium, which can obscure its onset in hospitalized patients. Variability in diagnostic criteria further complicates accurate recognition and prevalence estimates. This mini review summarizes current evidence on the prevalence, risk factors, and consequences of delirium in Parkinson’s disease (PD) and its associations with mortality and institutionalization. A total of 61 reports published between 1990 and 2025 were included across five domains: delirium diagnosis and prevalence, delirium subtypes, PD-specific delirium rates, risk factors and prevention, and treatment considerations. Foundational evidence reviews and official diagnostic documents (e.g., ICD-10, ICD-11, DSM criteria) were also incorporated as they remain internationally recognized standards for delirium diagnosis. Scholarly studies were appraised using the Joanna Briggs Institute (JBI) Critical Appraisal Checklists, with overall evidence quality judged to be moderate to high, while official diagnostic and guideline documents were considered high-quality based on their authoritative, consensus-driven development. Standardized clinical strategies for hospital management remain limited, but the available evidence supports the use of tailored approaches. We present evidence-based implications for clinical care aligned with the Parkinson’s Foundation Hospital Care Standards to improve recognition, prevention, and treatment. We emphasize the need for critical evaluation, methodological consistency, and acknowledgment of the dynamic care challenges posed by delirium in Parkinson’s disease.

## 1 Introduction

Parkinson’s disease (PD) is a neurodegenerative disorder affecting over 10 million people worldwide (Luo et al., 2024; Gerakios et al., 2024). Though most recognized by its motor symptoms, non-motor neuropsychiatric and cognitive impairment symptoms, such as deficits in attention, arousal, memory, language, and executive function, are also common in PD (Gerakios et al., 2024; Varadi, 2020). Delirium, often caused by medical, pharmacologic, and/or environmental factors, presents most commonly in older hospitalized patients, where predisposing conditions combined with hospital-related stressors can trigger the condition (Iglseder et al., 2022).

Though typically transient and reversible, delirium tends to be more prolonged and severe in individuals with Parkinson’s disease and cognitive impairment (PD-CI) (Gerakios et al., 2024). People with PD are also more likely to experience hypoactive or mixed delirium, often presenting as drowsiness or decreased alertness; such ambiguous symptoms can complicate timely diagnosis. Comorbid PD has been linked to an 11% increased odds of delirium during admission (George et al., 2023) and 88% increased odds of postoperative delirium.(Dham et al., 2023) The absence of validated screening/assessment tools responsive to the pathophysiological characteristics of PD contributes to challenges with diagnosis and distinguishing between delirium and PD-CI.

## 2 Methods

An evidence review committee was established, consisting of organizational professionals with expertise in PD program administration, research, and clinical practice standards. For the purposes of this review, Parkinson’s disease and cognitive impairment (PD-CI) is used as an umbrella term that encompasses mild cognitive impairment (MCI) associated with Parkinson’s disease (PD-MCI) as well as more advanced neurocognitive disorder due to PD (Parkinson’s disease dementia, PDD). Both PD-MCI and PDD increase vulnerability to delirium but distinguishing these medical concepts is important, as they differ in severity and clinical course.

The committee conducted a hand search of the literature published between 1990 and 2025, identifying studies relevant to the recognition, prevention, and management of delirium in PD. Reports were included if they addressed one or more of the following domains: (1) delirium diagnosis and prevalence, (2) delirium subtypes, (3) PD-specific delirium rates, (4) delirium risk factors and prevention, and (5) treatment considerations for delirium in PD. A total of 61 reports, including both scholarly studies and official diagnostic or guideline documents, met inclusion criteria.

Empirical studies were appraised using the Joanna Briggs Institute (JBI) Critical Appraisal Checklists according to study design (e.g., randomized controlled trial, cohort, cross-sectional, systematic review), with the overall quality judged to be moderate to high (Munn et al., 2020; Barker et al., 2023; Barker et al., 2023; Barker et al., 2025b; Barker et al., 2024; Barker et al., 2025a; Munn et al., 2021). Official documents [e.g., *International Classification of Diseases* (ICD), *Diagnostic and Statistical Manual of Mental Disorders* (DSM), and professional society guidelines (e.g., Parkinson’s Foundation and American Geriatrics Society)] were not subject to JBI appraisal but were considered high quality based on their authoritative and consensus-based development. A full summary of JBI appraisals for all included sources is provided in Supplementary Table S1. The committee engaged in virtual and email-based coordination to review and synthesize evidence related to the management of inpatient delirium in PD, consistent with the Parkinson’s Foundation Hospital Care Standards.

## 3 Results

A total of 61 reports published between1990 to the present were selected for inclusion. These included empirical studies (e.g., randomized controlled trials, cohort, cross-sectional, and systematic reviews) as well as official diagnostic and guideline documents. Overall, the body of evidence was judged to be of moderate to high quality based on structured appraisal, with official diagnostic references considered high quality as they were developed through international expert consensus. Findings are presented across five domains: (1) delirium diagnosis and prevalence, (2) delirium subtypes, (3) PD-specific delirium rates and outcomes, (4) risk factors and prevention, and (5) treatment considerations.

### 3.1 Delirium diagnosis and prevalence

The most commonly used tools to diagnose delirium are the *Diagnostic and Statistical Manual of Mental Disorders*, Fourth Edition (DSM-IV) and Fifth Edition, Text Revision (DSM-5-TR); the *International Classification of Diseases*, 10th Revision (ICD-10) and 11th Revision (ICD-11); and the *Confusion Assessment Method* (CAM) (American Pyshciatric Association, A.P. Delirium, 2022; Wei et al., 2008; Lawlor and Bush, 2014; Oldham and Weber, 2023; Meagher et al., 2014). The defining features of delirium for each tool are described in Table 1 [Lawson et al., 2019; WHO, 1992; Inouye et al., 1990]. Both the *Diagnostic and Statistical Manual of Mental Disorders* (DSM-IV and DSM-5-TR) and the International Classification of Diseases (ICD-10 and ICD-11) provide validated criteria for delirium to be used by healthcare professionals, while the CAM is a validated assessment tool designed to allow non-specialists such as nurses or admitting staff to identify delirium (Lawson et al., 2019), yet they are not consistently applied in routine clinical practice (Lawlor and Bush, 2014). Providers often rely on clinical judgment or other subjective measures to expedite patient intake or assessment, which may also contribute to diagnostic errors or underdiagnoses.

**Table 1:** Inpatient delirium detection in Parkinson’s disease: criteria, contexts, and clinical considerations.

Diagnostic tool	Core delirium definition (paraphrased)	Potential PD-related considerations	Common hospital care settings	Utility /limitations in PD
DSM-IV	Rapid onset disturbance in attention, awareness, and cognition; fluctuates; not explained by other neurocognitive disorders; physiologic cause identified.	Bradyphrenia, baseline cognitive slowing, and motor speech changes may obscure inattention; motor fluctuations may mimic severity changes.	Acute Med/Surg, ICU, Neuro Floor	Gold standard criteria; requires comprehensive assessment; not always practical for repeated bedside use.
DSM-5-TR	Disturbance in attention, awareness, and cognition that develops acutely, represents a change from baseline, and tends to fluctuate in severity during the day; the disturbance is a direct physiologic consequence of another medical condition, substance, or multiple causes.	DSM-5-TR emphasizes attention and awareness, which can be difficult to evaluate in PD due to bradyphrenia, slowed responses, and fluctuating alertness; overlap with PD dementia remains a diagnostic challenge.	Acute Med/Surg, ICU, Neuro Floor	Places greater emphasis on inattention and disturbances of awareness than the DSM-IV; Concordant with ICD-11 with ICD-11; requires careful differentiation from PD-related cognitive slowing and fluctuating motor function.
ICD-10	Characterized as F05 Delirium, not induced by alcohol or psychoactive substances, with concurrent changes in consciousness, attention, perception, thinking, memory, behavior, emotion, and sleep–wake cycles.	PD patients may have chronic sleep disruption and hallucinations; must differentiate from baseline PD dementia.	All inpatient settings	Broad definition; may over include baseline PD cognitive changes.
ICD-11	Acute onset of impaired attention, awareness, and cognitive disturbance, with a fluctuating course; disturbance results directly from an underlying medical condition, substance, or toxin, and is not better explained by another neurocognitive or mental disorder.	Overlap with Parkinson’s disease dementia or mild cognitive impairment may complicate recognition; psychosis, motor slowing, and sleep–wake cycle disruption common in PD may mimic or mask delirium features.	All inpatient settings	Provides updated, harmonized diagnostic criteria more consistent with DSM-5-TR; improves clarity on attention/awareness as core features; still requires clinical judgment to distinguish from baseline PD-related cognitive and motor fluctuations.
CAM	Requires acute onset and inattention, plus disorganized thinking or altered consciousness.	Motor slowness and hypophonia can be misinterpreted as altered consciousness; baseline fluctuation in PD dementia complicates assessment.	Bedside care in Med/Surg, Step-Down Units	Quick screen; widely used; requires familiarity with patient’s baseline.
MDAS	10-item clinician-rated scale assessing consciousness, orientation, attention, and psychomotor activity to quantify delirium severity.	Motor rigidity, bradykinesia, and hypophonia may confound scoring; PD-related baseline deficits can reduce specificity.	Acute Med/Surg, ICU, Neuro Floor	Reliable and validated severity measure; among best-performing scales for delirium detection in PD; requires trained staff and takes longer than CAM

DSM, Diagnostic and Statistical Manual of Mental Disorders; ICD, International Classification of Diseases; CAM, Confusion Assessment Method; MDAS, Memorial Delirium Assessment Scale; PD, Parkinson’s disease; PD-CI, Parkinson’s disease–cognitive impairment; ICU, Intensive Care Unit; Med/Surg, Medical-Surgical Unit.

Improving the accuracy and precision of diagnosing and characterizing inpatient delirium in PD may be supported by use of the Memorial Delirium Assessment Scale (MDAS), a 10-item clinician-rated scale that evaluates domains such as consciousness, orientation, attention, and psychomotor activity (Cullinan et al., 2023). The MDAS has demonstrated reliability in medically ill and neurologically vulnerable populations and is commonly used to quantify delirium severity. In PD, delirium severity has been assessed using the MDAS in clinical studies (Cullinan et al., 2023) and psychometric evaluation work in general medical cohorts has further confirmed its psychometric strength (Shyamsundar et al., 2009). The MDAS was recently regarded as one of the best performing tools for delirium detection in PD inpatients (Lawson et al., 2025).

The prevalence of delirium in inpatients, elderly or otherwise, is difficult to determine (Inouye et al., 2014). Measurement and hospital admission logistics [diagnostic tool used, admitting department—intensive care unit (ICU), emergency department (ED), etc.], influence case identification, making it challenging to capture and determine cases (Lawson et al., 2019). Estimates suggest that approximately half of all inpatients experience delirium during hospitalization, depending on age, reason for admittance, diagnostic tool used, and expertise of the staff administering it, among other variables (Inouye et al., 2014).

A meta-analysis covering 19,534 geriatric patients admitted to the ED found a pooled prevalence rate of 15.2% (Chen et al., 2022). A similar meta-analysis of 5,287 ICU patients (no age restriction) estimated a delirium prevalence of 33% (Wu et al., 2023). Higher estimated delirium prevalence rates came from a prospective study of elderly patients in Oman, with a 55.4% prevalence rate, missed by treating teams in 35.4% of patients (Al Farsi et al., 2023).

A recent cross-sectional estimation of delirium prevalence in the United States reported the results from the World Delirium Awareness Day survey. Ninety-one hospitals completed the survey at 8:00 a.m. (1,318 patients) and 8:00 p.m. (1,213 patients) detailing the prevalence of delirium within their wards, finding a prevalence rate of 16.4 and 17.9%, respectively.(Lindroth et al., 2024).

### 3.2 Hypoactive, hyperactive, and “mixed” delirium

Delirium has three diagnostic classifications: hypoactive, hyperactive, and “mixed” (an intermediate subtype). Hypoactive delirium is characterized by apathy, decreased speed of speech and alertness, unawareness, and reduced activity, while hyperactive delirium presents as increased activity, irritability, increased speed of speech, and hyper-alertness (Hayhurst et al., 2020). It is generally believed that the hyperactive subtype is less common than hypoactive, although studies have shown high heterogeneity in the ratios (Kumar et al., 2015; Meagher and Trzepacz, 1998; Camus et al., 2000).

Hyperactive delirium is more often found in patients with substance use disorders or those in trauma or intensive care settings, while hypoactive delirium is more often associated with psychotic disorders, diabetes, and infections (Kumar et al., 2015). Extended episodes of hypoactive delirium have also been independently associated with worse cognitive and executive outcomes months after the episode (Hayhurst et al., 2020).

No studies to date have investigated the incidence rates of these delirium subtypes in PD patients exclusively, and as such, caution should be used when extending the results of these studies to such a population. PD-CI or PD dementia may be misdiagnosed as hypoactive delirium or may mask hypoactive delirium in healthcare settings.

### 3.3 The PD impact on delirium rates

Accurate estimates of inpatient delirium prevalence are challenging to ascertain in individuals with PD compared to the general population due to overlapping cognitive symptoms and shared biological mechanisms that hinder the reliability of standard diagnostic tools. Many delirium diagnostic tools assess cognitive domains already impaired in PD, allowing delirium to be obscured by baseline PD-related deficits,(Lawson et al., 2022). The primary distinguishing feature between cognitive symptoms of PD and those of delirium is typically time; progressive, long-term changes suggest association with PD symptoms, whereas acute, fluctuating changes are characteristic of delirium (Daniels et al., 2024).

Because there are no validated tools for diagnosing delirium in people with PD, diagnosis of delirium in PD requires consistent, focused assessment with attention to health history and cognitive changes. However, most healthcare centers lack the resources or expertise to provide such assessments.

Several recent studies have attempted to estimate the amount of delirium underdiagnosis in patients with PD. One study followed 44 PD patients over 4 months, assessing prevalence and incidence of delirium across 53 hospital admissions (Lawson et al., 2019). Using DSM-5 criteria and patient history, researchers found a 34% prevalence and 56.6% incidence of delirium among PD inpatients. Symptoms were documented in 75% of cases, but only 37.9% received a formal diagnosis, often delayed. Hypoactive delirium was frequently missed, and just 11.5% of discharge summaries mentioned delirium (Cullinan et al., 2023).

A larger study followed 115 patients with PD and 199 older adult controls without PD admitted to a hospital in England, tracking delirium prevalence between the groups and monitoring rates of subsequent dementia, institutionalization, and mortality 12 months later (Gerakios et al., 2024). Delirium occurred in 67% of PD patients versus 39% of non-PD patients. Among those with PD, delirium was associated with threefold higher 12-month mortality, 6-fold higher dementia risk, and tenfold higher institutionalization, even after adjusting for age, sex, and frailty.

In the absence of validated tools, there is some evidence that certain bedside tests may be sensitive to detecting delirium in PD inpatients. However, the bedside tests most effective in identifying delirium in older adults without PD or dementia are different than those most effective for patients with PD. Combining both the attention rating from the Memorial Delirium Assessment Scale (MDAS) and arousal rating from the Glasgow Coma Scale (GCS) correctly classified delirium in 87% of PD patients in one study (Lawson et al., 2022).

Retrospective studies relying on hospital diagnoses without validated tools may lack reliability, while prospective research using standardized assessments and clinical history to distinguish PD-CI from delirium has been informative but limited (Gerakios et al., 2024). Despite gaps in prevalence data, existing evidence indicates that individuals with PD are at elevated risk for delirium, underscoring the need for heightened prevention and prompt treatment efforts (Cullinan et al., 2023).

### 3.4 Delirium risk factors and prevention methods

The incidence of delirium is influenced by both predisposing and precipitating factors. Predisposing factors (e.g., advanced age, pre-existing cognitive impairment, or sensory deficits) may increase a patient’s baseline vulnerability, while precipitating factors (e.g., infection, surgery, polypharmacy, or acute illness) represent acute events that can trigger the onset of delirium (Faeder et al., 2023). Although findings are mixed, emerging evidence suggests that PD may be an independent risk factor for delirium (Daniels et al., 2024; Lubomski et al., 2021; Boorsma et al., 2012). A potential bidirectional biological relationship may exist between PD-CI and delirium, with each acting as a predisposing risk factor for the other (Daniels et al., 2024). This parallels findings in dementia research, where dementia has been shown to confer a two- to five-fold increased risk of developing delirium (Fong et al., 2015).

Timely treatment remains a promising prevention measure, even in the ED. Among patients with PD seen in the ED, those who received a consultation-liaison (CL) pharmacy service used fewer injectable medications for agitation than those who did not receive CL services (Yuksel et al., 2024). Engaging care partners in monitoring and reporting changes in cognition can also support early identification of symptoms and help to prevent escalation (Shurer et al., 2023).

Multicomponent nonpharmacological approaches have been shown to be an effective way to prevent incidence of inpatient delirium. One of the most utilized programs is the Hospital Elder Life Program (HELP), which is designed to target the risk factors of cognitive impairment, sleep deprivation, immobility, visual impairment, hearing impairment, and dehydration (Inouye et al., 1999). During the patient’s stay, a multidisciplinary team of specialists and trained volunteers regularly conduct intervention activities including cognitively stimulating tasks, range-of-motion exercises, orientation check-ins, and hydration (Inouye et al., 1999).

The initial study for this intervention found that patients in the intervention group were 40% less likely to develop delirium (Inouye et al., 1999). However, such interventions were not significantly effective in preventing subsequent incidents of delirium. Additionally, a meta-analysis of 14 relevant studies found that multicomponent nonpharmacological intervention strategies such as HELP lowered the odds of developing delirium by 53% and reduced fall risk by 62%, the equivalent of preventing 4.26 falls per 1,000 patient-days (Hshieh et al., 2015).

Another well-established approach to delirium is called the ABCDEF bundle, for which the “D” stands for “Delirium: Assess, Prevent, and Manage” (Marra et al., 2017). This bundle is designed to help clinicians optimize ICU patient recovery, offering evidence-based recommendations on resource utilization and well-rounded care. The delirium component of the bundle describes the validated diagnostic tool to use to monitor delirium, the CAM-ICU, and offers guidance on delirium prevention. The ABCDEF bundle, along with other ICU healthcare best practices, was shown in two large studies to improve delirium outcomes with its increased use in hospital settings (Barnes-Daly et al., 2017; Pun et al., 2019).

Pharmacological prevention strategies for delirium prevention have shown mixed benefits (Daniels et al., 2024; Page et al., 2013; van den Boogaard et al., 2018; Chen, 2017). The most promising has been melatonin, with a meta-analysis finding that use in elderly patients in medical wards reduced incidence of delirium by 75% (Chen et al., 2016). However, the same significant effect was not seen in surgical ward patients. The melatonin receptor agonist ramelteon was also tested for its utility in reducing delirium incidence in patients undergoing surgery and found to be ineffective in a controlled trial (Oh et al., 2021). A small, randomized placebo-controlled trial was performed to test ramelteon on delirium prevention in elderly ICU patients and found it to provide a significant reduction in risk (Hatta et al., 2014), however caution has been raised about the interpretation of the study results due to small sample size and group imbalance (Perkisas and Vandewoude, 2015).

A retrospective database analysis found that PD is associated with increased odds of postoperative delirium across the 10 most common surgical procedures in the U. S. Compared to non-PD patients, those with PD also experience longer hospital stays, higher costs, and increased need for post-acute care, though postoperative mortality and use of life-sustaining interventions do not differ (Dham et al., 2023). In a separate study, intraoperative bispectral index (BIS) monitoring—an EEG-guided method to titrate anesthesia—reduced anesthetic use by 20–30% and lowered postoperative delirium risk by 35% (Chan et al., 2013). While this has not been studied specifically in PD, minimizing psychoactive drug exposure is likely beneficial in this population.

The American Geriatrics Society (AGS) Beers Criteria advises against the use of certain antipsychotics in older adults with PD due to the risk of severe extrapyramidal symptoms and cognitive decline (American Geriatrics Society 2012 Beers Criteria Update Expert Panel, 2012). The AGS recommendation aligns with PD specific recommendations within both the Parkinson’s Foundation Hospital Care Standards (PFHCS), the Parkinson’s Foundation Hospital Safety Guide, and the Age-Friendly Health Systems’ “4Ms” framework, which emphasizes avoiding high-risk medications like antipsychotics when possible, particularly in vulnerable populations such as those with PD (Campanelli, 2012; Parkinson's Foundation., Hospital Safety Guide Your Step-by-Step Resource for Better Parkinson’s Care in the Hospital, 2025; Parkinson's Foundation. Parkinson’s Foundation Hospital Care Recommendations, 2023).

The most effective methods to prevent delirium are nonpharmacological strategies that directly address the most significant risk factors (Zhao et al., 2023; Burton et al., 2021). The greatest challenge to implementing such measures broadly in healthcare settings is resourcing: intervention strategies like HELP require consistent and focused efforts with patients to maintain resistance to delirium (Hshieh et al., 2015; Hshieh et al., 2018). Low staffing and clinical capacity may not allow for such time and attention, even if delirium prevention could lead to reduced healthcare burden overall (Fong et al., 2023). Integrating Age-Friendly Health System measures, particularly the 4Ms framework (What Matters, Medication, Mentation, and Mobility), provides a structured approach to systematically address delirium risk within existing care model; this integration could involve embedding routine cognitive screening (Mentation), reviewing medications for delirium risk, promoting early mobility protocols, and aligning care with patient goals (What Matters) during interdisciplinary rounds (Institute for Healthcare Improvement, 2025).

### 3.5 Treatment of delirium and PD contraindications

The treatment of delirium in patients with PD begins with addressing the underlying conditions triggering delirium, such as inflammation, pain, or metabolic disturbances (Ebersbach et al., 2019). Though the pharmacological treatment of underlying conditions such as infections or pain is generally similar for patients with and without PD, individuals with PD are more vulnerable to neuropsychiatric side effects; therefore, drugs with psychoactive properties—such as fluoroquinolones (e.g., ciprofloxacin, levofloxacin), nitrofurantoin, and opioids—should be avoided or used with caution (Daniels et al., 2024).

Once the underlying cause of delirium has been identified and treated, the next step is to reduce or fully withdraw any routine medications that could potentially cause or worsen delirium. This is difficult for PD patients as the disease overlaps in several neurochemical ways to delirium, which results in routine PD medications having side effects that could exacerbate delirium.(Vardy et al., 2015) The recommended order in which to withdraw such deliriogenic anti-parkinsonian medications are as follows (Ebersbach et al., 2019):

1. Tapering off anticholinergics
2. Tapering off amantadine
3. Withdrawal of selegiline
4. Tapering off dopamine agonists and/or withdrawal of monoamine oxidase B (MAO-B) inhibitors
5. Withdrawal of catechol-O-methyltransferase (COMT) inhibitors and tapering off Levodopa

The steps outlined above for delirium management in individuals with PD should be approached cautiously and in consultation with a movement disorders specialist or neurologist, as recommended in the PFHCS, to ensure that treatment strategies do not exacerbate motor or cognitive symptoms (Institute for Healthcare Improvement, 2025).

When non-pharmacologic strategies are insufficient, appropriate psychoactive medications for PD patients may be used to manage agitation, hallucinations, or anxiety associated with delirium (Boettger et al., 2015). The PFHCS details the adverse effects of the use of inappropriate agents in individuals with PD and instead recommends safer alternatives that minimize motor and cognitive side effects (Parkinson's Foundation. Parkinson’s Foundation Hospital Care Recommendations, 2023). Additionally, some research has shown that typical antipsychotics increase the risk of mortality in patients with PD (Weintraub et al., 2016). As such, atypical antipsychotics should be considered first when pharmacological treatment is needed. Quetiapine has been found to have minimal motor deterioration side effects, but it has yet to be shown to have significant impact on psychosis symptoms (Desmarais et al., 2016).

Clozapine is a promising option, as one meta-analysis fully recommended its use in PD patients after finding it demonstrably effective and tolerable in that population (Frieling et al., 2007). Traditionally, this drug has had unique and cumbersome risks including the need to monitor white blood cell counts and requiring strict medication tracking due to the potential interactions clozapine may have with other antipsychotics (Ebersbach et al., 2019). However, the recent discontinuation of the U. S. Food and Drug Administration’s Clozapine Risk Evaluation and Mitigation Strategy program has eased many of the logistical burdens previously associated with its use (Palmer et al., 2025); This change may enable more timely and appropriate use of clozapine in managing psychosis and delirium, particularly in advanced PD, where the risk of untreated neuropsychiatric symptoms often outweighs the risks of the medication itself (Palmer et al., 2025).

Another therapeutic option for inpatient delirium management in PD is pimavanserin, a selective serotonin 5-HT2A receptor inverse agonist approved for the treatment of PD psychosis (Cummings et al., 2014). Unlike other antipsychotics, pimavanserin does not act on dopaminergic, histaminergic, or adrenergic receptors, making it less likely to worsen motor symptoms. Though its FDA approval is for hallucinations and delusions associated with Parkinson’s disease psychosis rather than delirium specifically, several reviews suggest it may be a promising candidate for managing delirium in PD due to its favorable motor safety profile (Tampi et al., 2019). However, data on its direct efficacy for delirium are limited, and use should be individualized, ideally in consultation with a movement disorders specialist (Seppi et al., 2019).

The use of benzodiazepines for delirium in people with PD is a debated matter, and there are no definitive studies investigating their efficacy in this context (Lonergan et al., 2009). In fact, benzodiazepines are strongly associated with the precipitation of delirium and as such are not recommended as an initial treatment choice for agitation except in cases of alcohol or sedative withdrawal (Irwin et al., 2013). Despite a lack of research supporting their utility in treating delirium, benzodiazepines are still a commonly administered drug for patients with hyperactive delirium (Neufeld et al., 2016). Ultimately, if the goal is to improve REM sleep for such patients, melatonin may be the better option (Chen et al., 2016).

Though nonpharmacological prevention remains the most effective strategy for managing delirium in patients with PD, evidence-based recommendations for medical treatment are available, as outlined in Table 2. Having an up-to-date list of medications is important for successful timely treatment and planning ahead, including putting together a preemptive plan with a healthcare professional on how to address delirium treatment if it were to occur could be a useful activity as recommended by the PFHCS for optimizing outcomes (Parkinson's Foundation. Parkinson’s Foundation Hospital Care Recommendations, 2023). Continued diagnostic monitoring for delirium after the initial case is also critical, especially for chronic and complex conditions like PD, as the rate of subsequent incidence is higher than that for the initial occurrence (Gerakios et al., 2024; Lawson et al., 2025; Aikawa et al., 2024; Green et al., 2021).

**Table 2:** Medication considerations in Parkinson’s disease patients with delirium: integrated guidance from beers criteria, PF hospital recommendations, delirium subtype, and stage.

Medication type	Beers criteria (2023) Guidance focus area	PF hospital guidelines (2021)	Delirium type	Delirium stage
Benzodiazepines / Z-drugs	Avoid for insomnia or agitation; increased risk of confusion, falls, cognitive decline	Avoid; may worsen confusion and impair mobility in PD	Hyperactive, Mixed	Onset, Advancement
Sedating antihistamines	Strong anticholinergic burden; avoid in older adults	Avoid; exacerbates confusion, sedation, worsens mobility	Hypoactive, Mixed	Onset, Advancement
Typical antipsychotics (e.g., haloperidol)	Avoid due to extrapyramidal effects, sedation, anticholinergic load	Contraindicated in PD; may cause motor crisis or worsening	Hyperactive	Peak, Exacerbation
Atypical antipsychotics (e.g., quetiapine)	Use with caution; appropriate in select cases for behavioral disturbances	Preferred for psychosis if needed; clozapine if quetiapine ineffective and labs monitored	Hyperactive, Mixed	Exacerbation, Stabilization
Pimavanserin	Not included in Beers Criteria; selective serotonin inverse agonist (5-HT2A antagonist) designed for PD psychosis	May be considered in PD psychosis where quetiapine/clozapine are not tolerated or contraindicated; minimal motor side effects	Hyperactive, Mixed	Exacerbation, Stabilization
Tricyclic antidepressants	Avoid due to sedation, orthostatic hypotension, and anticholinergic effects	Avoid; may worsen cognition and cause sedation	Hypoactive	Onset, Advancement
SSRIs (e.g., sertraline)	Acceptable; monitor for hyponatremia, GI upset	Preferred antidepressants in PD	Hypoactive	Stabilization, Recovery
Opioids	Use with caution; increased risk of delirium, sedation, constipation	Avoid unless essential; prefer acetaminophen	All types (worsens arousal)	Onset, Exacerbation
Anticholinergics (e.g., oxybutynin, benztropine)	Strongly discouraged; increased delirium risk, cognitive decline	Avoid; worsens both motor and cognitive symptoms	Hypoactive	Onset, Advancement
Dopamine agonists	Caution: associated with hallucinations, sleep disturbance, confusion	Use sparingly; may worsen delirium symptoms	Hyperactive, Mixed	Onset, Exacerbation
Amantadine	CNS effects (e.g., confusion, hallucinations); caution in older adults	May worsen psychosis and confusion; avoid if possible	Hyperactive, Mixed	Advancement, Exacerbation
Melatonin	Safe and preferred for insomnia	First-line for sleep issues in PD	Hypoactive	Onset, Advancement
Levodopa	Safe and appropriate for PD	Essential; continue on time to avoid motor decline and confusion	All types	All stages (essential at all times)

PD, Parkinson’s disease; PF, Parkinson’s Foundation; CNS, central nervous system; SSRI, selective serotonin reuptake inhibitor; 5-HT2A, 5-hydroxytryptamine 2A (serotonin) receptor; GI, gastrointestinal; RBD, REM sleep behavior disorder; REM, rapid eye movement; EPS, extrapyramidal symptoms; QoL, quality of life; FDA, U. S. Food and Drug Administration; REMS, Risk Evaluation and Mitigation Strategy; Beers Criteria, American Geriatrics Society Beers Criteria for Potentially Inappropriate Medication Use in Older Adults.

## 4 Conclusion

People with PD are at high risk for delirium, yet no validated diagnostic tests exist, limiting clarity and precision in treatment decisions. Prevention should therefore be prioritized, using nonpharmacological strategies and monitoring tailored to individual symptoms. In this way, PD symptoms may be differentiated from emerging delirium symptoms in a timely manner, potentially reducing patient and healthcare burden. Delirium identification should be addressed promptly, including medication adjustments and consultations with neurologists. Maintaining an up-to-date list of PD prescriptions and tolerabilities supports optimal care in emergencies, where preparation and advocacy can help prevent delirium.
