# Supplementary material for: Improving recognition and management of inpatient delirium in Parkinson’s disease: evidence review and implications for clinical care
Source: Front Aging Neurosci. 2025 Oct 29;17:1693827. doi: 10.3389/fnagi.2025.1693827 (PMC12605173; doi:10.3389/fnagi.2025.1693827)
Supplement: Supplementary file 1 [file Table_1.DOCX]

**Table S1. JBI Critical Appraisal of Included Studies and Documents**

| **Reference (First Author, Year)** | **Study Type/Focus** | **Delirium Care Domain(s)** | **JBI Checklist Used** | **Appraisal Outcome** |
| --- | --- | --- | --- | --- |
| Inouye, 1990 | Diagnostic tool development (CAM validation) | Diagnosis & Prevalence | Analytical Cross-Sectional; Prevalence Studies | High |
| World Health Organization (WHO), 1992 | Diagnostic guideline (ICD-10) | Diagnosis & Prevalence | N/A (Guideline) | High (authoritative) |
| Meagher, 1998 | Phenomenology / review of delirium | Delirium Subtypes | Systematic Reviews and Research  Syntheses | Moderate |
| Inouye, 1999 | Randomized controlled trial (HELP) | Risk Factors & Prevention | Checklist for Randomized Controlled Trials | High |
| Camus, 2000 | Subtype profiles (observational) | Delirium Subtypes | Analytical Cross-Sectional | Moderate |
| Frieling, 2007 | Structured review & meta-analysis (clozapine in PD) | Treatment & Contraindications | Systematic Reviews and Research  Syntheses | High |
| Lonergan, 2009 | Cochrane review (benzodiazepines for delirium) | Treatment considerations for delirium in PD | Systematic Reviews and Research  Syntheses | High |
| Boorsma, 2012 | Cohort (nursing homes/residential care) | Delirium risk factors and prevention | Cohort Studies | Moderate |
| American Geriatrics Society Panel, 2012 | Medication appropriateness guideline (Beers Criteria) | Risk Factors & Prevention | N/A (Guideline) | High (authoritative) |
| Chan, 2013 | RCT (BIS-guided anesthesia) | Risk Factors & Prevention | Checklist for Randomized Controlled Trials | Moderate |
| Irwin, 2013 | Expert recommendations (delirium management) | Risk Factors & Prevention | Systematic Reviews and Research  Syntheses | High (expert consensus) |
| Page, 2013 | RCT (haloperidol in ICU) | Treatment & Contraindications | Checklist for Randomized Controlled Trials | Moderate |
| Cummings et al., 2014 (Lancet) | Randomized controlled trial | Treatment considerations | Checklist for Randomized Controlled Trials | High |
| Hatta, 2014 | RCT (ramelteon prevention) | Risk Factors & Prevention | Checklist for Randomized Controlled Trials | Moderate |
| Inouye, 2014 | Narrative review (delirium in elderly) | **Diagnosis & Prevalence; Risk Factors & Prevention** | Systematic Reviews and Research  Syntheses | Moderate |
| Boettger, 2015 | Comparative management (observational) | Treatment & Contraindications | Cohort Studies | Moderate |
| Fong, 2015 | Review (delirium–dementia interface) | **Diagnosis & Prevalence; Risk Factors & Prevention** | Systematic Reviews and Research  Syntheses; Prevalence Studies | Moderate |
| Hshieh, 2015 | Systematic review/meta-analysis (multicomponent non-pharm) | Risk Factors & Prevention | Systematic Reviews and Research  Syntheses | High |
| Kumar, 2015 | Observational (hyperactive vs hypoactive) | Delirium Subtypes | Analytical Cross-Sectional Studies | Moderate |
| Perkisas, 2015 | JAMA Network Clinical Review/Education (ramelteon) | **Risk Factors & Prevention; Treatment & Contraindications** | Text & Opinion | Moderate |
| Vardy, 2015 | Review (delirium in PD) | Treatment & Contraindications | Text & Opinion / Narrative Review | Moderate |
| Chen, 2016 | Meta-analysis (melatonin for prevention) | Treatment & Contraindications | Systematic Reviews and Research  Syntheses | High |
| Desmarais, 2016 | Systematic review (quetiapine in PD psychosis) | Treatment & Contraindications | Systematic Reviews and Research  Syntheses | Moderate–High |
| Neufeld, 2016 | Systematic review/meta-analysis (antipsychotics for delirium) | Treatment & Contraindications | Systematic Reviews and Research  Syntheses | High |
| Weintraub, 2016 | Cohort (antipsychotics & mortality in PD) | Treatment & Contraindications | Cohort Studies | High |
| Barnes-Daly, 2017 | Cohort / QI (ABCDEF bundle across hospitals) | Risk Factors & Prevention | Cohort Studies | Moderate–High |
| Chen, 2017 | Review (psychosis treatment in PD) | Treatment & Contraindications | Systematic Reviews and Research  Syntheses | Moderate |
| Marra, 2017 | Review (ABCDEF bundle in critical care) | Risk Factors & Prevention | Systematic Reviews and Research  Syntheses | Moderate |
| Youn, 2017 | Review (delirium in PD or related) | PD-specific Rates/Outcomes; Treatment & Contraindications | Systematic Reviews and Research  Syntheses | Moderate |
| Hshieh, 2018 | Systematic review/meta-analysis (HELP effectiveness) | Risk Factors & Prevention | Systematic Reviews and Research  Syntheses | High |
| van den Boogaard, 2018 | RCT (REDUCE haloperidol) | Treatment & Contraindications | Checklist for Randomized Controlled Trials | High |
| Ebersbach, 2019 | Review (management of delirium in PD) | Treatment & Contraindications | Systematic Reviews and Research  Syntheses | Moderate |
| Lawson, 2019 | Systematic review (defining delirium in PD) | Diagnosis & Prevalence; PD-specific Rates/Outcomes | Systematic Reviews and Research  Syntheses; Prevalence Studies | High |
| Pun, 2019 | Large cohort (ICU liberation collaborative, ABCDEF) | Risk Factors & Prevention | Cohort Studies | High |
| Seppi, 2019 | Evidence-based medicine review (nonmotor symptom management updates) | Non-motor treatment, treatment considerations | Systematic Reviews and Research  Syntheses | High |
| Tampi, 2019 | Narrative review | Treatment considerations | Systematic Reviews and Research  Syntheses | Moderate |
| Hayhurst, 2020 | Cohort (delirium subtype outcomes) | Delirium Subtypes | Cohort Studies | High |
| Burton, 2021 | Cochrane review (non-pharm prevention, non-ICU) | Risk Factors & Prevention | Systematic Reviews and Research  Syntheses | High |
| Green, 2021 | Cohort (first delirium episode in PD/parkinsonism) | PD-specific Rates/Outcomes | Cohort Studies | High |
| Lubomski, 2021 | Review (QoL PD patients/caregivers) | PD-specific Rates/Outcomes | Systematic Reviews and Research  Syntheses | Moderate |
| Oh, 2021 | RCT (RECOVER ramelteon postoperative) | Risk Factors & Prevention | Checklist for Randomized Controlled Trials | Moderate |
| Chen, 2022 | Prevalence Review, Emergency Departments | Diagnosis & Prevalence | Systematic Reviews and Research  Syntheses; Prevalence Studies | High |
| Lawson, 2022 | Cross-sectional (bedside attention/arousal tests) | Diagnosis & Prevalence | Analytical Cross-Sectional; Prevalence Studies | High |
| Al Farsi, 2023 | Prospective cohort (medical wards) | Diagnosis & Prevalence; Risk Factors & Prevention | Cohort Studies | Moderate–High |
| Cullinan, 2023 | Prospective cohort (PD admissions) | PD-specific Rates/Outcomes | Cohort Studies | High |
| Dham, 2023 | Retrospective database study (postoperative delirium & PD) | PD-specific Rates/Outcomes | Cohort Studies | High |
| Faeder, 2023 | Review (prevention & treatment in older adults) | Risk Factors & Prevention; Treatment & Contraindications | Systematic Reviews and Research  Syntheses | Moderate |
| Fong, 2023 | Implementation/Program evaluation (remote HELP) | Risk Factors & Prevention | Cohort Studies | Moderate |
| Parkinson’s Foundation, 2023 | Clinical guideline (Hospital Care Recommendations) | Treatment & Contraindications | N/A (Guideline) | High (authoritative) |
| Shurer, 2023 | Qualitative (patient-centered PD hospital needs) | PD-specific Rates/Outcomes; Risk Factors & Prevention | Checklist for Qualitative Research | Moderate–High |
| Wu, 2023 | Systematic review/meta-analysis (ICU delirium) | Risk Factors & Prevention | Systematic Reviews and Research  Syntheses | High |
| Zhao, 2023 | Overview of systematic reviews (non-pharm P/T) | Risk Factors & Prevention | Systematic Reviews and Research  Syntheses | High |
| Daniels, 2024 | Review (diagnostic challenges in advanced PD) | Diagnosis & Prevalence; PD-specific Rates/Outcomes | Systematic Reviews and Research  Syntheses | Moderate |
| Gerakios, 2024 | Prospective cohort (PD vs controls outcomes) | PD-specific Rates/Outcomes | Cohort Studies | High |
| Lindroth, 2024 | Cross-sectional (US prevalence survey, WDAD) | Diagnosis & Prevalence | Analytical Cross-Sectional; Prevalence Studies | High |
| Yuksel, 2024 | Retrospective cohort (ED carbidopa-levodopa continuation) | Risk Factors & Prevention | Cohort Studies | Moderate |
| American Psychiatric Association | Diagnostic guideline (ICD-11) | Diagnosis & Prevalence | N/A (Guideline) | High (authoritative) |
| Parkinson’s Foundation, 2025 | Guideline/Guide (Hospital Safety Guide) | Treatment & Contraindications | N/A (Guideline) | High (authoritative) |
| Lawson, 2025 | Diagnostic accuracy (bedside tools in PD) | Diagnosis & Prevalence | Analytical Cross-Sectional / Prevalence Studies | High |
| Palmer, 2025 | Perspective/Policy note (Clozapine REMS discontinuation) | Treatment & Contraindications | Text & Opinion | Moderate |
| Institute for Healthcare Improvement Improvement, n.d. | Framework/Measure (Age-Friendly 4Ms) | Risk Factors & Prevention | N/A (Framework) | High (authoritative) |

**Abbreviations:** **ABCDEF**, Awakening and Breathing Coordination, Delirium monitoring/management, Early exercise/mobility, and Family engagement bundle; **BIS**, Bispectral Index; **CAM**, Confusion Assessment Method; **DSM**, Diagnostic and Statistical Manual of Mental Disorders; **ED**, Emergency Department; **GCS**, Glasgow Coma Scale; **HELP**, Hospital Elder Life Program; **ICD**, International Classification of Diseases; **IHI**, Institute for Healthcare Improvement; **JBI**, Joanna Briggs Institute; **MAO-B**, Monoamine Oxidase B; **MDAS**, Memorial Delirium Assessment Scale; **PD**, Parkinson’s disease; **QoL**, Quality of Life; **QI**, Quality Improvement; **RCT**, Randomized Controlled Trial; **REMS**, Risk Evaluation and Mitigation Strategy; **WHO**, World Health Organization; **WDAD**, What Disturbs the Aging Delirium study.

**Note:** Randomized controlled trials (RCTs) were rated *High* when they clearly described randomization and allocation concealment, maintained appropriate blinding (participants, providers, and/or outcome assessors), achieved complete or near-complete follow-up, and used valid and reliable outcome measures. RCTs with limitations such as small sample size, high attrition, or insufficient detail on randomization/blinding were rated *Moderate*. Systematic reviews and meta-analyses were rated *High* when they followed rigorous methods, such as PRISMA or Cochrane standards, with comprehensive search strategies, transparent inclusion/exclusion criteria, formal quality appraisal of included studies, and appropriate synthesis methods. Reviews with methodological limitations, incomplete reporting, or restricted scope were rated *Moderate*. Cohort, cross-sectional, and qualitative studies were rated *High* when they used clear inclusion criteria, reliable and validated outcome measures, and adequately addressed or acknowledged potential confounding; they were rated *Moderate* when one or more of these elements were insufficiently described or absent. Narrative and text/opinion reviews were generally rated *Moderate*, given the inherent limitations of the design, provided they referenced and synthesized evidence from high-quality empirical studies.
